# Added value of multiple autoantibody testing for predicting progression to inflammatory arthritis in at-risk individuals

**DOI:** 10.1136/rmdopen-2022-002512

**Published:** 2022-12-19

**Authors:** Frederique Ponchel, Laurence Duquenne, Xuanxiao Xie, Diane Corscadden, Farag Shuweihdi, K Mankia, L A Trouw, Paul Emery

**Affiliations:** 1 Leeds Institute of Rheumatic and Musculoskeletal Medicine, University of Leeds, Leeds, UK; 2 Leeds Institute of Health Sciences, Faculty of Medicine, University of Leeds, Leeds, UK; 3 Department of Immunology, Leiden University Medical Center, Leiden, Netherlands; 4 Leeds NIHR Biomedical Research Centre, Leeds Teaching Hospitals Trust, Leeds, UK

**Keywords:** Arthritis, Rheumatoid, Anti-Citrullinated Protein Antibodies, Rheumatoid Factor

## Abstract

**Background:**

Predicting progression to clinical arthritis in individuals at-risk of developing rheumatoid arthritis is a prerequisite to developing stratification groups for prevention strategies. Selecting accurate predictive criteria is the critical step to define the population at-risk. While positivity for anti-citrullinated protein antibodies (ACPA) remains the main recruitment biomarker, positivity for other autoantibodies (AutoAbs) identified before the onset of symptoms, may provide additional predictive accuracy for stratification.

**Objective:**

To perform a multiple AutoAbs analysis for both the prediction and the time of progression to inflammatory arthritis (IA).

**Methods:**

392 individuals were recruited based on a new musculoskeletal complaint and positivity for ACPA or rheumatoid factor (RF). ELISAs were performed for ACPA, RF, anti-nuclear Ab, anti-carbamylated protein (anti-CarP) and anti-collagen AutoAbs. Logistic and COX regression were used for analysis.

**Results:**

Progression to IA was observed in 125/392 (32%) of cases, of which 78 progressed within 12 months. The AutoAbs ACPA, RF, anti-CarP were individually associated with progression (p<0.0001) and improved prediction when combined with demographic/clinical data (Accuracy >77%; area under the curve (AUC) >0.789), compared with prediction using only demographic/clinical data (72.9%, AUC=0.760). Multiple AutoAbs testing provided added value, with +6.4% accuracy for number of positive AutoAbs (AUC=0.852); +5.4% accuracy for AutoAbs levels (ACPA/anti-CarP, AUC=0.832); and +6.2% accuracy for risk-groups based on high/low levels (ACPA/RF/anti-CarP, AUC=0.837). Time to imminent progression was best predicted using ACPA/anti-CarP levels (AUC=0.779), while the number of positive AutoAbs was/status/risk were as good (AUC=0.778).

**Conclusion:**

We confirm added value of multiple AutoAbs testing for identifying progressors to clinical disease, allowing more specific stratification for intervention studies.

**What is already known about this subject?**

- Rheumatoid arthritis is now recognised as a continuum of disease with a preclinical phase identified by the presence of Autoantibodies highlighting a breach of tolerance. Both rheumatoid factor and anti-citrullinated protein antibodies have been associated with progression to inflammatory arthritis from this at-risk, preclinical stage.

**What does this study add?**

- Other autoantibodies have been described but have not yet been assessed for their additional value in improving prediction models. Whether combination of autoantibodies may have added value also remains to be investigated.

**How might this impact on clinical practice or further developments?**

- Being able to predict the highest risk of progression with better accuracy and/or the imminence of it, would allow the design of clinical trials testing interventions aiming at preventing progression to disease, notably by shortening the follow-up time needed to observe progression.

## Introduction

Rheumatoid arthritis (RA) is a chronic disease with substantial impact on the lives of people worldwide, with >1 million patients having severe disability from the disease in Europe alone. Despite the recent progress achieved with ‘treat-to-target’ therapeutic strategies^1^ and the earlier access to treatment enabled by the European Alliance of Associations for Rheumatology (EULAR)-2010 diagnostic criteria,^2^ the outcome of RA is still a major concern as well as a financial burden on health services, patients and society with considerable socioeconomic cost.^3–5^

Over a decade ago, research highlighted a preclinical phase of the disease with the recognition of an inflammatory arthritis continuum (IAC),^6^ moving the knowledge gap to the earliest stages of disease progression. Although the exact pathogenesis of RA remains unclear, autoimmune processes are believed to play a role and recent research has proposed a series of events underpinning the IAC.^6^ A breach of tolerance needs to occur (ie, development of autoimmunity) and this assumption has largely been verified by the presence of specific autoantibodies (AutoAb), years before the development of symptoms,^7–11^ occurring in association with an major histocompatitbility complex/T-cell signalling genetic background.^12 13^ Systemic autoimmunity is not sufficient to initiate clinical arthritis and a second series of events (the second hit) needs to happen, for which the cells and the possible triggers are not yet known, although several hypotheses have been proposed (implicating cytokines, netosis, pain, osteoclast and/or T-cell or Th17 cells).^14–18^ This understanding offers an unprecedented opportunity to study ‘at-risk’ individuals and ultimately intervene with the ambition of preventing the progression to arthritis by means of life-style modification or pharmacotherapy.

Several risk stratification models have been developed including demographic, genetic, clinical, imaging, serological and immunological biomarkers with variable predictive value.^19–26^ Reported predictive biomarkers however have low sensitivity for the prediction of progression to disease stages while specificities are relatively high (>75%). Other studies have reported potential biomarkers, while not modelling for the actual outcome.^8 10 11 27–30^

The presence of disease specific AutoAbs (notably anti citrullinated protein antibodies, ACPA) prior to disease development remains the best-known risk-factor,^7 8^ although it is also the main biomarker used to recruit most at-risk cohorts, (including arthralgia and first degree relationship to RA patients). Different ACPA tests also showed different results (positivity and levels), based on the repertoire of peptides used in second and third generation kits.^31^ Other AutoAbs have been detected years before the onset of inflammatory symptoms including anti-carbamylated protein (anti-CarP), rheumatoid factor (RF) (of the IgM class, further referred to as RF), anti-native and anti-glycated collagen-II AutoAbs.^9–11^

In this study, we aimed to investigate the added value of testing for the presence of multiple AutoAbs for the prediction of progression to clinical synovitis, in a cohort of at-risk individuals selected on the basis of positivity for ACPA and/or RF associated with a new onset of non-specific musculoskeletal symptoms. Several AutoAbs were explored, notably using in-house ELISA for anti-CarP and anti-collagen AutoAbs. Our data suggest added-value for ACPA, RF, anti-CarP and anti-collagen-II AutoAbs when tested individually either as positive/negative status, continuous levels or dichotomised as high-risk/low-risk groups. Detecting positivity of multiple AutoAbs improved predictive accuracy, which may facilitate more precise selection of study populations as well as determination of high risk of progression in a short time frame to prioritise intervention.

## Methods

### Subjects and samples

Serum samples were obtained from participants attending an at-risk research clinic at Chapel Allerton Hospital in Leeds between 2008 and 2018.

Briefly, individuals aged ≥18 years, with a new non-specific musculoskeletal joint symptom presenting to their primary care physician or other health professional were referred as previously described.^22 23^ A new musculoskeletal symptom was defined as pain in any joint and/or musculoskeletal symptom (including but not limited to rotator cuff tendonitis, subacromial bursitis, carpal tunnel syndrome, tendonitis, or epicondylitis), which was not previously reported. Individuals were then recruited to attend the research clinic if they tested positive for ACPA or RF as tested by National Health Service (NHS) services as described below. Exclusion criteria were previous diagnosis of an inflammatory condition, exposure to DMARDs and presence of a swollen joint at first visit. Participants were followed 3 monthly for 1 year and then yearly, until progression to inflammatory arthritis (IA) developed (ie, clinical swelling of at least one joint), evaluated by a rheumatologist/rheumatology research nurse. Progression could occur at any time, and patients had open access to immediate appointment if experiencing a new symptoms. Classification for RA criteria were recorded at study visit only and progression to RA was observed although later during follow-up.

The dataset used in this report was frozen in late 2019 and do not include visits, samples or data collected over the pandemic. We only included participants up to the point in time for which they had visit data. The strategy for data analysis was to use (1) the exact time of progression for participants who progressed at any time during follow-up; (2) for non-progressors, we only included those having at least 12-month follow-up (up to 10 years), while follow-up duration was calculated from baseline until their last available appointment.

A serum sample was collected in clotting tube at inclusion in the cohort, spun for 10 min, no shorter than 30 min and no longer than 1 hour after collection, aliquoted and stored at −30°C.

### Autoantibody status and levels

In the absence of absolute standards for AutoAbs assays, arbitrary units (AU) or optical densities (OD) values were used to describe levels observed. Hospital services were used to initially assess positivity (yes/no) for ACPA and/or RF.

RF levels (IgM) were measured by nephrology and considered positive above a cut-off of 20 AU/mL. Anti-nuclear Ab (ANA) were detected as part of our routine assessment and detected by immunofluorescence (homogeneous, nucleolar or specked staining) and reported as negative/positive. Levels of ACPA and RF measured in 15 HCs were all below cut-off and ANA staining were all negative.

ACPA IgG levels were then measured using a seconnd-generation research ELISA (CCP-2, immunoCap Phadia). Cut-off for positivity was set according to manufacturers at 10 AU/ml. Patients with borderline results were retested at 3 months follow-up (mostly confirming negative).

In-house ELISA was developed for IgG autoantibodies for Anti-CarP and anti-native and anti-glycated collagen. In addition, IgG-RF was measured using a commercial ELISA (EuroImmune, Germany) as per manufacturer’s instruction (cut-off 20 RU/mL).

Anti-CarP Abs were measured in the Leiden University Medical Centre (Leiden, The Netherland) as previously described,^10^ using carbamylated foetal calf serum (10 µg/mL) as antigen. Positivity for Anti-CarP Abs in HC (n=175, median age 51, range 27–65) was previously described^32 33^ and a cut-off set at 235 UA/mL.

In-house ELISA for the detection of anti-native collagen (CI and CII) and glycated collagen (GLY-CI and GLY-CII) was performed as previously described.^11 33^ Briefly, collagen CI (Cellsystems) and CII (MD Biosciences) were chemically modified by Maillard reaction in a single batch to generate post-translationally modified GLY-CI/GLY-CII. ELISA plates were coated with 10 µg/mL of GLY-CI/GLY‐CII or native-CI/CII. ELISA OD values were recorded following normalisation to BSA and GLY-BSA (10 µg/mL). Ranges of results in HC were previously established^33^ (n=98, median age 51, range 27–65) and set at 1.8 and 3 OD/mL for native CI/CII, and 2 and 2.4 for GLY-CI/GLY-CII respectively.

Enough serum was available from only for 152 and 71 individuals for RF-IgG and the collagen related AutoAb testing respectively. ANA were tested in 290 participants. We had missing data for a few samples for the main three autoAbs tested. This affected mainly RF with 11 missing data (status and levels). For ACPA and anti-CarP, this affected 29 (levels only) and 20 cases, respectively (status and level).

### Statistical analysis

Frequencies were compared using Pearson’s χ^2^ test. Numerical variables were not normally distributed (Kolmogorov-Smirnov test) and compared using non-parametric Mann‐Whitney U tests. A value of p<0.05 was considered statistically significant. Where needed, corrections for multiple testing were applied.

Missing data frequencies ranged from none to 31/451 (6.9%) for both clinical and AutoAbs data. Multiple data imputation was performed using five cycles. Logistic regressions were used to model the added value of multiple AutoAb testing for the prediction of progression, using a stepwise Forward approach. A Cox regression was also used to model time to progression. Data were presented using GraphPad Prism V.8 and analysed using SPSS V.26 and /or R V.4.1.2 packages.

## Results

### Population description

Individuals at-risk of RA, followed since 2008 and up to December 2019, was included in our AutoAbs analysis, when progression to IA could be established over at least 12 months of follow-up, excluding recently recruited patients with less than 12 months of follow-up data, withdrawal by choice. Patients with whom we lost contact (over 12 year) were included to the last appointment available. This allowed us to include 392 participants. Participants had a median age of 51 years (IRQ 18 years, range 18–82) and 70% were women. Participant’s demographic and clinical characteristics (including missing data) according to their progression status are shown in table 1.

**Table 1:** Overall cohort clinical description (n=392)

	Univariate statistics				Unadjusted		Adjusted model (logistic regression)		Adjusted model (COX regression)	
	Missing data n/451	Progressor n=125	Non-progressor n=267	P value	OR (95% CI)	P value	OR (95% CI)	P value	HR (95% CI)	P value
Age* (years)	None	53 (21) (22–80)	50 (19) (18–82)	0.108	1.018 (0.998 to 1.030)	0.030				
Sex (F/M)	None	89/36	195/72	0.718	0.940 (0.638 to 1.577)	0.081				
Smoking (never/ever)	1	36/89	122/144	<0.0001	3.072 (1.994 to 4.733)	<0.0001	2.626 (1.718 to 4.331)	<0.0001	1.813 (1.228 to 3.106)	0.015
HLA SE (yes/no)	1	93/32	121/145	<0.0001	3.239 (2.102 to 4.990)	<0.0001	3.204 (1.902 to 4.832)	<0.0001	2.575 (1.522 to 3.976)	<0.0001
EMS* (minutes)	1	30 (60) (0–480)	5 (30) (0–560)	<0.0001	1.003 (1.000 to 1.006)	0.031				
ESR* (minutes)	1	14 (19) (1–83)	10 (15) (1–71)	<0.0001	1.042 (1.018 to 1.051)	<0.0001	1.040 (1.015 to 1.050)	<0.0001	1.031 (1.015 to 1.044)	<0.0001
CRP (hs)* (mg/L)	1	2.6 (8) (0–80)	1.5 (4) (0–83)	0.015	1.037 (0.009 to 1.064)	0.011				
TJC78*	1	2 (4) (0–21)	1 (3) (0–16)	0.002	1.138 (1.045 to 1.199)	0.001	1.162 (1.064 to 1.243)	<0.0001	1.135 (1.066 to 1.199)	<0.0001
Family relatives (yes/no)	None	37/88	80/187	0.778			Accuracy 72.9% SEN 37% (31–47) SPE 89% (85–92) PPV 63% (53–71) NPV 75% (72–77) AUC 0.760 (0.709 to 0.811)		AUC 0.725 (0.723 0.727)	
Diabetes (yes/no)	12	20/105	33/234	0.325						

*Median (IQR) (range).

AUC, area under curve; CRP, C reactive protein (high sensitivity); EMS, early morning stiffness; ESR, erythrocyte sedimentation rate; PPV/NPV, positive/negative predictive value; SE, share epitope; SEN, sensitivity; SPE, specificity; TJC78, tender joint count in 78 joints.

We observed progression to clinical IA in 125/392 (32%). The median time of progression was 12 months (IQR 21 months and range 1–113) and the follow-up in non- progressors had a median time of 38 months (IQR 38 and range 12–144). Progression over years is illustrated in online supplemental figure 1. Age was not different between progressors and non-progressors but female gender tended to be more frequent in progressors (p=0.108). A patient-reported case of first degree arthritis in the family was also not associated with progression. Previously reported risk factors from this cohort^22^ were associated with progression, including smoking history, early morning stiffness, erythrocyte sedimentation rate (ESR), C reactive protein and painful joint counts (all p<0.001). Comorbidities such as diabetes were not associated with progression.

10.1136/rmdopen-2022-002512.supp1 Supplementary data

There was randomness of missing data at low frequencies (maximum 5.1% missing data excluding RF-IgG, ANA and anti-collagen, see below), therefore, missing data were imputed for both clinical as well as for AutoAbs. Unadjusted OR for each variable were calculated from the pooled 5 cycles of imputation and were not significantly different from the original dataset.

### AutoAbs determination

Because of limited amount of serum, all AutoAbs could not be tested in all samples (missing tests detailed in table 2), notably for the anti-collagen AutoAbs and RF-IgG, while ANA were not performed routinely in 36% of participants, resulting in 7 AutoAbs tested.

**Table 2:** AutoAbs individual predictive performance (n=392)

(A) AutoAbs status						
	**Univariate** **p value**	**OR (95% CI)**		**AUC** **(95% CI)**	**SEN/SPE** **(95% CI)**	**PPV/NPV** **(95% CI)**
ACPA 29 missing	<0.0001	10.95 (6.20 to 19.35)		0.748 (0.699 to 0.798)	86% (79 to 92) 62% (59 to 67)	52% (48 to 56) 90% (86 to 93)
RF eleven missing	<0.0001	4.80 (3.03 to 7.57)		0.687 (0.621 to 0.734)	68% (60 to 75) 68% (62 to 73)	51% (45 to 55) 81% (77 to 85)
anti-CarP 20 missing	<0.0001	4.54 (2.88 to 7.18)		0.670 (0.610 to 0.730)	55% (45 to 63) 74% (69 to 80)	53% (46 to 59) 76% (63 to 72)
anti-native CII 71 cases	0.111					
anti-GLY-CII 71 cases	0.075					
(B) AutoAbs levels						
	**Univariate** **p value**	**OR (95% CI)**	**Cut-off (AU/mL)**	**AUC (95% CI)**	**SEN/SPE** **(95% CI)**	**PPV/NPV** **(95% CI)**
ACPA 29 missing	<0.0001	1.005 (1.004 to 1.006)	150	0.770 (0.716 to 0.824)	56% (46 to 66) 75% (70 to 80)	43% (37 to 50) 84% (80 to 86)
RF eleven missing	<0.0001	1.002 (1. to 1.003)	60	0.702 (0.645 to 0.750)	5% (1 to 8) 98% (95 to 99)	54% (21 to 38) 67% (66 to 68)
Anti-CarP 20 missing	<0.0001	1.001 (1.001 to 1.003)	300	0.720 (0.666 to 0.775)	26% (19 to 35) 95% (92 to 97)	70% (57 to 80) 72% (67 to 75)
(C) Risk categories						
	**Univariate** **p value**	**OR (95% CI)**		**AUC (95% CI)**	**SEN/SPE** **(95% CI)**	**PPV/NPV** **(95% CI)**
ACPA 29 missing	<0.0001	6.230 (3.971 to 9.776)		0.710 (0.649 to 0.766)	61% (51 to 69) 80% (75 to 85)	60% (53 to 66) 81% (77 to 84)
RF eleven missing	<0.0001	4.020 (2.617 to 6.176)		0.641 (0.579 to 0.702)	52% (43 to 60) 78% (73 to 83)	54% (47 to 61) 77% (74 to 80)
Anti-CarP 20 missing	<0.0001	4.169 (2.695 to 6.449)		0.675 (0.615 to 0.735)	51% (42 to 59) 80% (75 to 85)	57% (50 to 63) 76% (73 to 79)

ACPA, anticitrullinated protein antibody; AUC, area under the curve; PPV/NPV, positive and negative predictive value; RF, rheumatoid factor; SEN/SPE, sensitivity/specificity.

**Figure 1:**
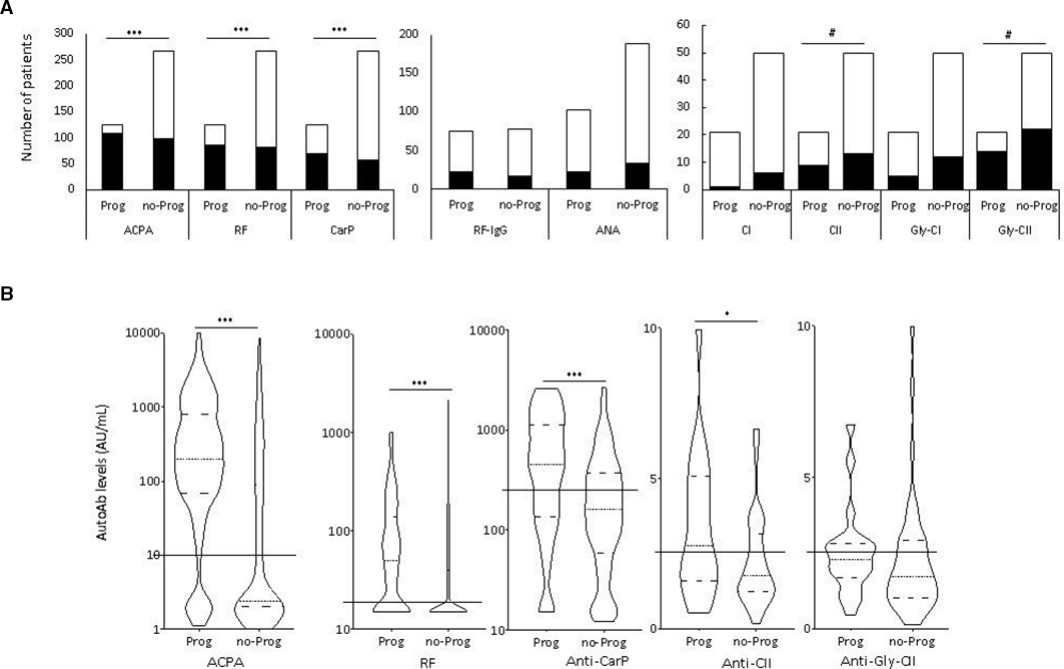
Positivity (A), as number of patients and (B) levels of AutoAb in at-risk individuals. (A) Bars represent the number of individual tested positive (black) or negative (white) for each individual. X^2^ tests were used individually to assess associations with progression (***p<0.0001, #p<0.100). ACPA n=363, RF n=381, anti-CarP n=372, RF-IgG n=152, ANA n=290, all anti-collagen autoAbs n=71. (B) Violin plots represent the distribution of AutoAb levels observed. Solid lines across the plot indicate the positivity cut-off for each test. Medians and quartiles of distribution are indicated by dotted and dashed lines within violin plot respectively. MWU tests comparing levels were used individually to assess associations with progression (*p<0.05, ***p<0.001, #p<0.100). ACPA, anticitrullinated protein antibody; ANA, anti-nuclear Ab; RF, rheumatoid factor.

Most participants were ACPA+ (206/392, 52%), accounting for individual with initial borderline levels, found negative on retesting, and 61 were ACPA+only. 167 participants were RF+ (figure 1A, median 65 UA/mL, IQR 140 UA/mL) of which, 39 participants were RF+only. RF-IgG and ANA were positive in 50% and 20% of samples tested respectively. Positivity for anti-CarP was observed in 126/372 (33.9%) of participants, 10 being anti-CarP+only. Anti-native-CI and CII AutoAbs were detected at low frequencies (9.9% and 29.6%, respectively) while anti-GLY-CII (50.7%) was more frequent but not anti-GLY-CI (24%).

Using positivity status (figure 1A), ACPA, RF and anti-CarP were highly associated with progression (table 2A, all p<0.0001). Although the assays could only be performed in a small number of samples (n=71), anti-GLY-CII and anti-native-CII showed a trend for association with progression (p=0.075 and p=0.111, respectively). RF-IgG, ANA and Anti-CI AutoAbs were not associated with progression.

Using continuous levels of AutoAbs (figure 1B), there was significantly higher levels of ACPA (irrespective of the test used), RF and anti-CarP AutoAbs in progressors (table 2B, p<0.0001), while levels were significant higher for anti-native CII (p=0.048) but not for anti-GLY-CII.

Results presented below account for the cohort of 392 patients following imputations for ACPA, RF and anti-CarP, while RF-Ig and ANA AutoAbs were not pursued further due to lack of association with progression and anti-collagen AutoAbs as it could only be tested in 71 patients.

### Prediction of progression: individual AutoAbs

**Figure 2:**
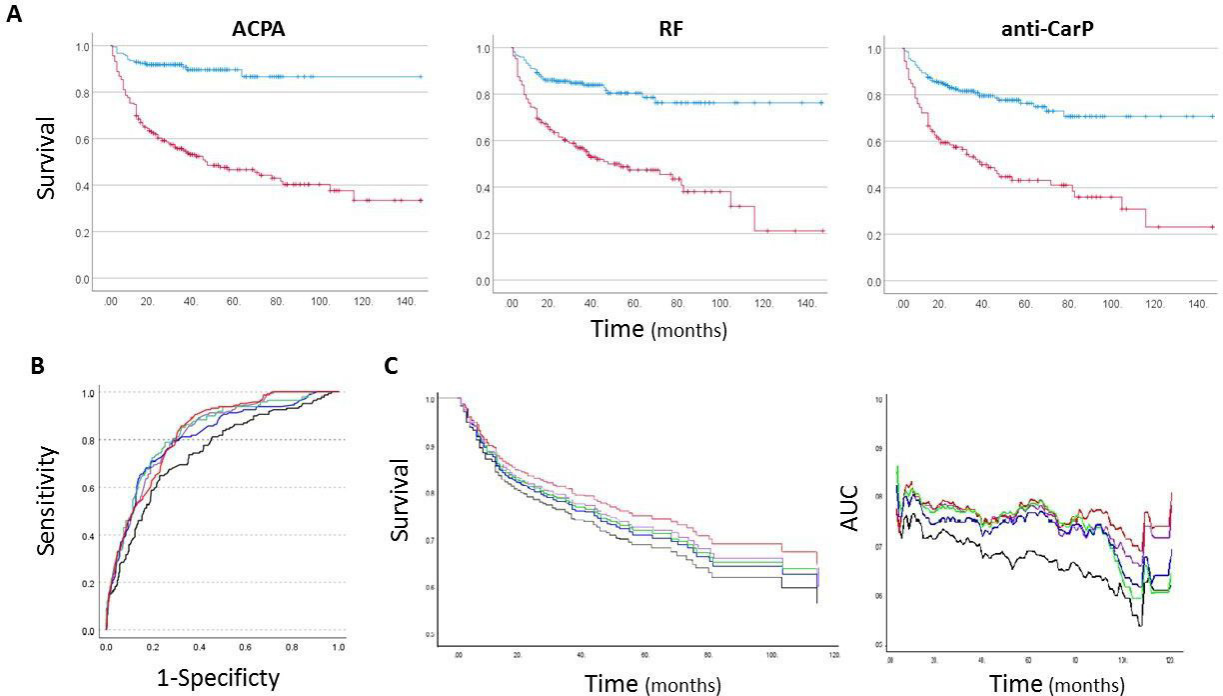
Modelling the predictive value of autoAbs in at-risk individuals. (A) Individual time of progression with respect to positivity for the three main autoAbs tested (red positive, blue negative). (B) Logistic regression: AUC for prediction models using clinical data and multiple autoAbs based on status, levels, high and low risk groups or autoAbs count. (C, D) Cox regression: Survival and AUC for prediction models using clinical data and multiple autoAbs based on status, levels, risk groups or autoAbs count. (B–D) Black clinical data only; green clinical data+status; red clinical data+levels; blue clinical data+risk group; purple clinical data+autoAbs count. ACPA, anticitrullinated protein antibody; AUC, area under the curve; RF, rheumatoid factor.

Altogether, ACPA, RF, anti-CarP and anti-CII AutoAbs were taken forward, while RF-IgG, ANA, and CI AutoAbs were no longer included in the analysis as not associated with progression. The rate of progression in ACPA+, RF+ and anti-CarP+individual are illustrated in figure 2A, confirming clear separation between status.

ACPA positivity performed best, the OR being 10.95 (table 2A), with best sensitivity (86%) and 90% negative predictive value (NPV) notably compared with RF (81%) and anti-CarP (76%). RF and anti-CarP had high OR>4.5 compared with other variable (table 1).

An analysis performed on continuous AutoAbs levels (table 2B) confirmed the predictive performance of the different AutoAbs with area under the curve (AUC) values ranging from 0.702 for RF to 0.770 for ACPA. This allowed for levels to be dichotomised for a more practical high versus a low risk of progression, using a cut-off set at 80% specificity for progression. Individual predictive performances for each AutoAbs were calculated for high vs low risk (table 2C). ORs were not better than when using AutoAbs status but AUC were improved for anti-CarP (0.675) while but not for ACPA or RF. All positive predictive value (PPV) were relatively similar (range 54%–60%), while NPV were best for CCP-res assays (81%).

AUC is individually presented for ACPA/RF/anti-CarP in online supplemental figure 2 for status, levels and risk groups.

### Prediction of progression: added-value of Individual autoAbs

To demonstrate the possible added value of AutoAbs testing, we first established which demographic and clinical variables were predictive of progression (from eight parameters routinely recorded). A regression model built from the clinical data retained 4-variables (table 1, AUC=0.760 and displayed in online supplemental figure 3), including a genetic risk factor (shared epitope SE), an environmental risk factor (smoking), pain (tender joint count in 78 joints, TJC78) and an inflammation marker (ESR) which reproduced previously reported results^22^ and allowed 72.9% of individual’s outcome to be predicted correctly (Accuracy) and all four variable being highly predictive (all p<0.0001). This 4-variables model was considered the reference for further comparison.

We evaluated the added value of AutoAbs individually (table 3 and online supplemental figure 3) for status (A), levels (B) and risk categories (C). All models retained the four original clinical variables with each AutoAb, suggesting that they all had individual added value, improving accuracy (+4.1% to +4.4%) over the reference model and better AUC (+0.029 to +0.076). Overall, the AutoAb status with the best prediction accuracy were ACPA or anti-Carp (7.3%), best specificity-PPV was with anti-Carp (90% and 70%) although the AUC=0.789 was less good than ACPA (0.836) or RF (0.800).

**Table 3:** Added-value of individual autoAbs (n=392)

(A)	+ACPA status		+RF status		+Anti CarP status	
	OR (95% CI)	P value	OR (95% CI)	P value	OR (95% CI)	P value
Smoking	1.842 (1.18 to 3.215)	<0.0001	2.372 (1.538 to 4.012)	<0.0001	2.412 (1.552 to 3.981)	<0.0001
SE	2.606 (1.533 to 4.137)	0.006	3.084 (1.791 to 4.713)	<0.0001	2.767 (1.711 to 4.418)	<0.0001
ESR	1.032 (1.007 to 1.045)	<0.0001	1.031 (1.007 to 1.043)	0.005	1.033 (1.009 to 1.045)	0.004
TJC78	1.167 (1.063 to 1.262)	<0.0001	1.173 (1.074 to 1.268)	<0.0001	1.157 (1.057 to 1.239)	<0.0001
AutoAb	7.820 (3.937 to 12.048)	<0.0001	3.935 (2.2666 to 5.663)	<0.0001	3.198 (1.706 to 4.219)	<0.0001
Accuracy	77.3%		77%		77.3%	
SEN SPE	60% (50–67) 84% (79–88)		52% (43–59) 89% (85–93)		50.5% (39–55) 90 (84–92)	
PPV NPV	66% (58–71) 82% (77–84)		68.5% (61–77) 80% (76–81)		70% (59–72) 79% (75–80)	
AUC (95% CI)	0.836 (0.793 to 0.879)		0.800 (0.753 to 0.846)		0.789 (0.740 to 0.839)	
(B)	**+ ACPA levels**		**+ RF levels**		**+ anti CarP levels**	
	**OR (95% CI)**	**P value**	**OR (95% CI)**	**P value**	**OR (95% CI)**	**P value**
Smoking	2.122 (1.375 to 3.613)	0.001			2.439 (1.479 to 3.978)	<0.0001
SE	2.752 (1.605 to 4.239)	<0.0001			3.109 (1.849 to 4.818)	<0.0001
ESR	1.037 (1.010 to 1.047)	0.002			1.031 (1.011 to 1.053)	0.003
TJC78	1.195 (1.082 to 1.272)	<0.0001			1.150 (1.056 to 1.234)	0.002
AutoAb	1.005 (1.004 to 1.007)	<0.0001			1.001 (1.001 to 1.002)	<0.0001
Accuracy	77.6%		No added value		77.7%	
SEN SPE	52% (42–58) 89.5% (85–92)				46.5% (35–51) 92% (87–94)	
PPV NPV	70% (58–74) 79% (76–82)				73.5% (61–78) 77.5% (74–79)	
AUC (95% CI)	0.824 (0.780 to 0.868)				0.787 (0.736 to 0.838)	
(C)	**+ ACPA risk group**		**+ RF risk group**		**+ Anti CarP risk group**	
	**OR (95% CI)**	**P value**	**OR (95% CI)**	**P value**	**OR (95% CI)**	**P value**
Smoking	2.060 (1.378 to 3.644)	<0.0001	2.2735 (1.478 to 3.802)	0.002	2.378 (1.498 to 3.841)	<0.0001
SE	2.822 (1.672 to 4.459)	<0.0001	3.208 (1.850 to 4.756)	<0.0001	2.761 (1.701 to 4.116)	<0.0001
ESR	1.041 (1.011 to 1.048)	0.001	1.033 (1.009 to 1.045)	0.001	1.032 (1.007 to 1.044)	0.003
TJC78	1.202 (1.091 to 1.283)	<0.0001	1.156 (1.060 to 1.240)	<0.0001	1.144 (1.050 to 1.226)	0.002
AutoAb	6.906 (4.012 to 11.890)	<0.0001	2.770 (1.641 to 4.677)	<0.0001	3.741 (2.200 to 6.361)	<0.0001
Accuracy	78.3%		75.8%		78.3%	
SEN SPE	57% (46–63) 89% (85–92)		42% (34–50) 91% (87–94)		49.5% (38–55) 92% (86–93)	
PPV NPV	70% (62–77) 81% (76–83)		69% (60–77) 77% (73–78)		74% (61–77) 79.5% (75–80)	
AUC (95% CI)	0.832 (0.789 to 0.875)		0.770 (0.718 to 0.813)		0.792 (0.728–0.823)	

ACPA, anticitrullinated protein antibody; AUC, area under thecurve; ESR, erythrocyte sedimentation rate; PPV/NPV, positive/negative predictive value; RF, rheumatoid factor; SE, share epitope; SEN, sensitivity; SPE, specificity; TJC78, tender joint count in 78 joints.

For continuous levels (table 3B), ACPA and anti-CarP but not RF, demonstrated added value over the reference model. The best performing model was with anti-CarP with+5.5% accuracy but the best AUC was for ACPA (0.824).

Using AutoAbs risk categories (table 3C), the best models were similar for ACPA or anti-Carp (+5.4% accuracy) but the best AUC was for ACPA (0.832). All model had similar specificity (anti-CarP being the best with 92%) while ACPA had highest sensitivity (57%).

### Added value of multiple autoantibody testing

We used combination ACPA with RF and anti-CarP to evaluate the added value of multiple AutoAbs testing. A progression rate is presented in online supplemental figure 4 with respect to combined positivity for the three autoAbs.

First, Status were combined in a single variable, counting the number of positive AutoAbs. Data suggested significant association of higher number of positive AutoAbs with progression (p<0.0001) with triple negativity (123/392 (28% of the cohort) poorly associated with progression (5/123 (4.0%)), 1 AutoAb+ (117/392 (30% of cohort)) showing increased progression rate (28/117 (23%)), 2 AutoAbs+ (83/392 (21% of cohort)) a further increase (44/83 (53%)) and triple positivity (69/392 (17.6% of the cohort)) the highest progression rate (48/63 (76%). The number of positive AutoAbs had an individual OR=3.422 and AUC=0.813.

The AutoAbs count was then analysed with the variables of the reference model to address its added-value (table 4A, figure 2B). The combination provided a clear improvement on accuracy (+6.4%) and AUC=0.852). To account more for the importance of each AutoAbs to the prediction, models were then constructed using status, levels and risk groups. Using status, the model retained both ACPA and RF but removed anti-CarP. It increased slightly the prediction (77.8% accuracy) of the models with only ACPA or anti-Carp (table 3, 77.3%), while showing an increased AUC=0.855, but not better specificity over anti-Carp-alone (90%). There was added accuracy (+5.4%) of multiple AutoAbs testing using continuous levels keeping ACPA and anti-CarP above the model using anti-CarP levels alone (77.7%), as well as improved AUC=0.832. Using risk categories, the model retained again ACPA and anti-CarP, and showed good accuracy (79.1%) although slightly bellow AutoAb counts (−0.2%) but above autoAb status (+1.3%).

**Table 4:** Added value of multiple autoAb testing: logistic and COX regression T(est of proportional hazards (PH) models overtime were valid (p>0.05) for all predictors)

(A) Logistic regression, n=392								
	Counting autoAbs		AutoAb model (status)		AutoAb model (levels)		AutoAb model (risk)	
	OR (95% CI)	P value	OR (95% CI)	P value	OR (95% CI)	P value	OR (95% CI)	P value
Smoking	1.806 (1.100 to 3.034)	0.037	1.772 (1.121 to 3.089)	0.045	2.180 (1.268 to 3.345)	0.003	1.959 (1.129 to 3.052)	0.015
SE	2.340 (1.519 to 4.166)	<0.0001	2.495 (1.531 to 4.224)	0.002	2.894 (1.678 to 4.484)	<0.0001	2.608 (1.612 to 4.381)	<0.0001
ESR	1.024 (1.073 to 1.275)	0.033	1.025 (1.003 to 1.047)	0.024	1.004 (1.002 to 1.006)	0.006	1.036 (1.014 to 1.059)	0.001
TJC78	1.167 (1.073 to 1.275)	0.002	1.178 (1.080 to 1.292)	0.001	1.171 (1.075 to 1.264)	<0.0001	1.188 (1.081 to 1.272)	<0.0001
AutoAbs count	2.898 (2.126 to 3.465)	<0.0001						
ACPA			6.751 (3.550 to 11.039)	<0.0001	1.004 (1.002 to 1.005)	<0.0001	5.487 (3.095 to 9.728)	<0.0001
RF			3.220 (1.959 to 5.104)	<0.0001				
Anti-CarP					1.001 (1.000 to 1.001)	0.006	2.156 (1.196 to 3.888)	0.011
Accuracy SEN SPE PPV NPV AUC (95% CI) p value	79.3% 70% (61–79) 82.5% (77–86) 60% (53–66) 88% (84–91) 0.852 (0.813–0.891) <0.0001		77.8% 67.5% (49–66) 85% (78–88) 60% (56–70) 86.5% (77–83) 0.855 (0.817–0.893) <0.0001		78.3% 73% (63–82) 80% (75–84) 50% (44–57) 92% (88–94) 0.832 (0.789–0.875) <0.0001		79.1% 71% (61–79) 82.5% (77–86) 59% (52–65) 88% (85–91) 0.837 (0.797–0.880) <0.0001	

(B) Cox regression for imminent progression (next 12 months), n=344								
	Counting autoAbs		AutoAb model (status)		AutoAb model (levels)		AutoAb model (risk)	
	HR (95% CI)	P value	HR (95% CI)	P value	HR (95% CI)	P value	HR (95% CI)	P value
SE	2.409 (1.430 to 3.751)	0.001	2.729 (1.428 to 3.044)	<0.0001	2.671 (1.424 to 3.075)	<0.0001	2.304 (1.450 to 4.100)	0.002
ERS	1.017 (1.082 to 1.215)	0.043			1.024 (1.008 to 1.050)	0.004	1.026 (1.010 to 1.044)	0.002
EMS	1.003 (1.001 to 1.005)	0.005	1.003 (1.001 to 1.005)	0.010	1.002 (1.001 to 1.004)	0.043		
TJC78	1.107 (1.082 to 1.215)	0.003	1.132 (1.065 to 1.200)	<0.0001	1.120 (1.044 to 1.198)	0.001	1.155 (1.078 to 1.238)	<0.0001
AutoAb count	2.217 (1.803 to 2.789)	<0.0001						
ACPA			4.441 (2.342 to 6.323)	<0.0001	1.003 (1.002 to 1.004)	<0.0001	3.589 (2.372 to 6.086)	<0.0001
RF			2.754 (1.500 to 3.060)	<0.0001				
Anti-CarP					1.001 (1.000 to 1.003)	<0.0001	1.778 (1.076 to 2.766)	0.025
AUC (95% CI) p value	0.778 (0.776 0.781) <0.0001		0.778 (0.776 0.781) <0.0001		0.779 (0.777 0.782) <0.0001		0.778 (0.777 0.782) <0.0001	
Nagelkerke R^2^	0.285		0.273		0.287		0.269	

ACPA, anticitrullinated protein antibody; AUC, area under the curve; EMS, early morning stiffness; ESR, erythrocyte sedimentation rate; PPV/NPV, positive/negative predictive value; RF, rheumatoid factor; SE, share epitope; SEN, sensitivity; SPE, specificity; TJC78, tender joint count in 78 joints.

We then performed an analysis of time to progression using a COX regression (table 4B). We chose to look at imminent progression within 12 months which did not include patients who progressed late (n=344, as illustrated in online supplemental figure 1). The proportional hazards assumption of Cox model was checked and found satisfied (p>0.05). The reference model selected the different variables (SE, ESR EMS and TJC78) and had an AUC=0.725 (table 1). The COX models did not retain smoking but showed clear increase in AUCs (table 4B, figure 2C). The status and risk category models kept ACPA and RF and were as good as counting autoAbs model (all same AUC=0.778). The best improvement in AUC was observed for AutoAbs levels (AUC=0.779, figure 2D) with ACPA and anti-CarP, suggesting value for utilising levels for predicting time of imminent progression.

## Discussion

Multiple AutoAbs have been associated with RA, including triple positivity for ACPA/RF/anti-CarP^34 35^ and more recently with its pre-clinical stages.^7–11^ Here, we show the individual and combined value of AutoAbs used for RA classification for the prediction of progression to clinical disease. In this large cohort of 392 individuals selected on the basis of having a new non-specific musculoskeletal complain and being ACPA+and/or RF+, progression to IA was associated with ACPA, RF, anti-CarP and trends for anti-collagen-II Abs (likely due to small numbers), while not with anti-collagen-I and ANA. These associations were individually predictive improving the performance of the prediction based solely on demographic and clinical characteristics.^22^ Levels (although not for RF) consistently provided higher specificity and better AUC, and PPV, while sensitivity was more variable and NPV were similar. Here, the combination of AutoAbs levels with demographic and clinical data showed improvement over an individual AutoAb model, while dichotomising levels into high/low risk groups was better than using positive status.

Our data aligned with a meta-analysis of 12 studies,^36^ showing evidence of the value of triple positivity, ACPA, RF and anti-CarP, in identifying individuals at-risk from the healthy population (however not including demographic and clinical data in the models). We further demonstrate that triple positivity as well as analysis by status, levels or risk groups all suggested clear added value of using anti-CarP AutoAb for a better prediction of progression overall as well as its timing.

Participants included in our cohort based on NHS-ACPA positivity showed a high rate of false positive compared with research ELISA test (also previously observed).^31^It may be beneficial to use a higher cut-off (at 10 AU/mL for example) for recruitment of individual with non-specific musculoskeletal symptoms. This would allow >75% of false positive cases, not to be referred for clinical follow-up. On the other hand, these individuals still present with a new MSK pain complaint and may actually offer an interesting ACPA-negative research control group. This had notably allowed for RF+only participants (n=39, 8 progressed) and anti-CarP+only (n=10, 1 progressed) participants to be identified (see online supplemental figure 2 for illustration). Altogether, 123 participants had a triple negative status and five progressed (1.2%) compared with 26/110 (23.6%) in the group with at least one auto-Ab positive (p<0.0001). Importantly for the 186 ACPA-negative participant 14 of the 17 that progressed were either RF+ (n=9) or anti-CarP+ (n=1) or both (n=4), leaving three progressors currently not identified by an autoantibody (although one was positive for anti-Gly-CII+).

Currently, many biomarker have been proposed in individual at-risk of RA. From a single blood test, our study has confirmed the previously reported better individual predictive value of ACPA (using second or third generation tests,^37 38^ while still demonstrating that combinations of autoantibodies are more informative that each autoantibody alone. In addition to serological testing, other biomarkers such as high resolution imaging (ultrasound and MRI)^39–41^ or T-cell subsets^23 24^ or the combination of some, may still provide increased predictive value. The impact of certain biomarkers may also be greatest in those with ‘imminent’ arthritis, given most individuals develop subclinical joint and tendon inflammation prior to the onset of clinical joint swelling^41^ while other may provide additional value for understanding pathogenesis.

Limitations to the study, despite a large number of participants was the uniqueness of recruitment criteria, which currently limits any replication. There are different recognised populations considered at-risk of RA, notably as most recently redefined by a EULAR taskforce.^42^ These include seropositive arthralgia, clinically suspected arthralgia (CSA), first degree relatives of RA patients and ACPA+individuals with non-specific MSK symptoms. In Leeds, we chose to recruit the later, as at-risk individuals often initially present to primary care clinicians while CSA requires specialist assessment (ie, secondary care). Furthermore, there is a much higher overall rate of progression in these individual than reported in CSA, while ACPA+positive RA is usually more severe compared with seronegative disease.^43 44^ As criteria are needed to define an at-risk population and to balance the specificity of the recruitment with the number of cases needed to develop models. The overall cohort tested over 9000 potential participants since 2008, while about >750 were included being ACPA+in line with reported prevalence of ACPA-positivity.^45^ The populations and models developed for ACPA+arthralgia^22 46^ are therefore not directly transferable to cohort of patients with CSA (as defined by EULAR taskforce^42 47 48^), or in first degree relatives of patients with IA,^49–51^ which are the other main characteristics used for selecting at risk individuals. In addition, progression rate in these different groups are different and if 32% for IA in our cohort, progression to RA was only observed following from the development of IA in less than 25% of cases.

In conclusion, our data confirm the value of multiple AutoAbs testing for three particular antigens in preclinical RA, while suggesting that others may also have value (anti-collagen-II AutoAbs notably) and allowing for the exclusion of some (ANA, RF-IgG, anti-Collagen-I but not II). A baseline assessment of multiple AutoAbs may, therefore, inform a follow-up strategy for individual complaining of non-specific musculoskeletal symptoms. A research strategy based on an observational design may direct participants for 3 monthly vs annual review, using an overall prediction of progression (logistic models) based on counting AutoAbs or using risk groups. Alternatively, a strategy aiming at modifying the risk of progression (ie, intervention) may be better tested using a stratification based on time to progression (Cox regression) reducing the need for long-term follow-up.

## Data Availability

No data are available.
