# Supplementary material for: Added value of multiple autoantibody testing for predicting progression to inflammatory arthritis in at-risk individuals
Source: RMD Open. 2022 Dec 19;8(2):e002512. doi: 10.1136/rmdopen-2022-002512 (PMC9764647; doi:10.1136/rmdopen-2022-002512)

Supp-Figure 1 : Progression rate in groups of patients with 1, 2, 3, 5 or more years of follow-up

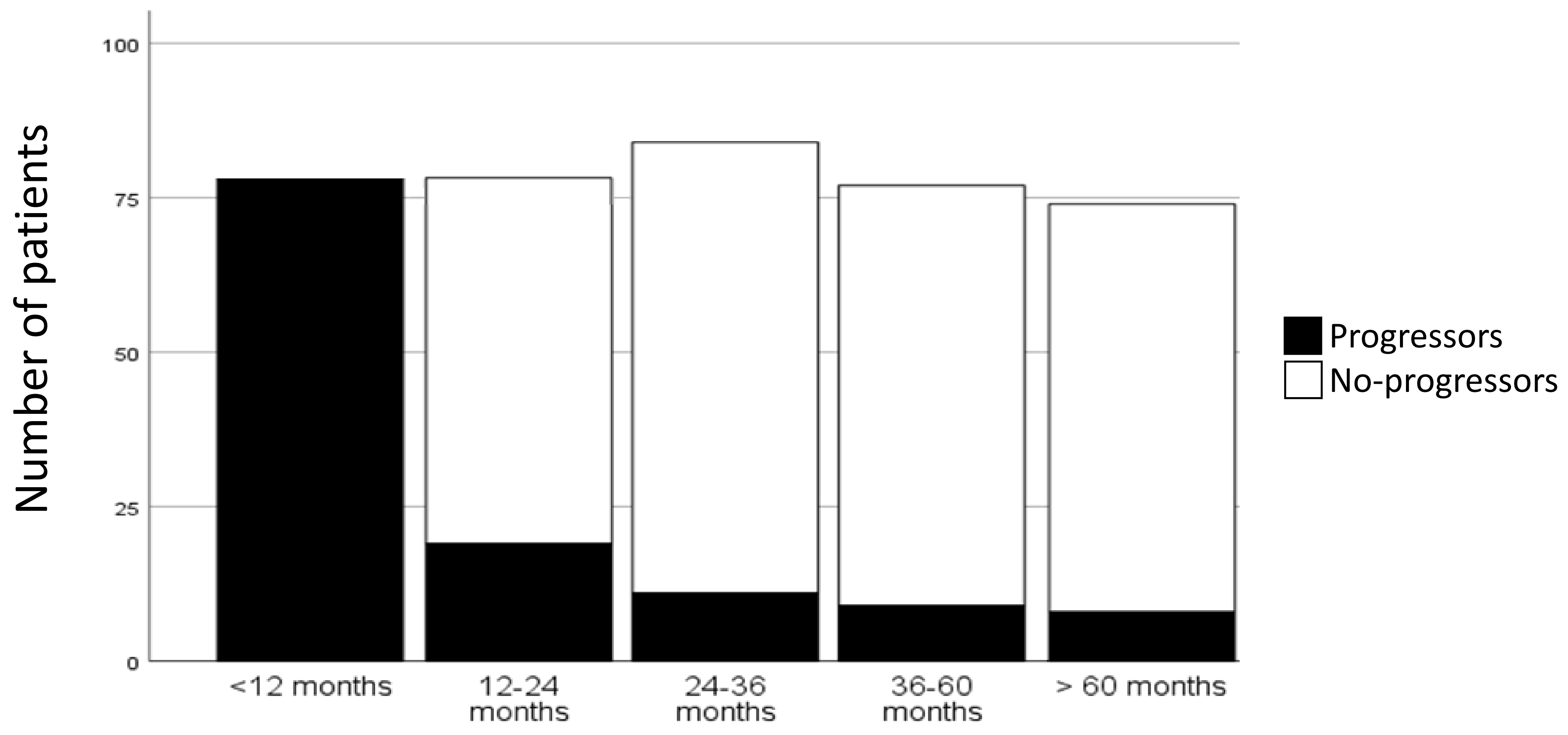

Supp-Figure 2 Individual value of the autoAbs for predicting progression (AUC)

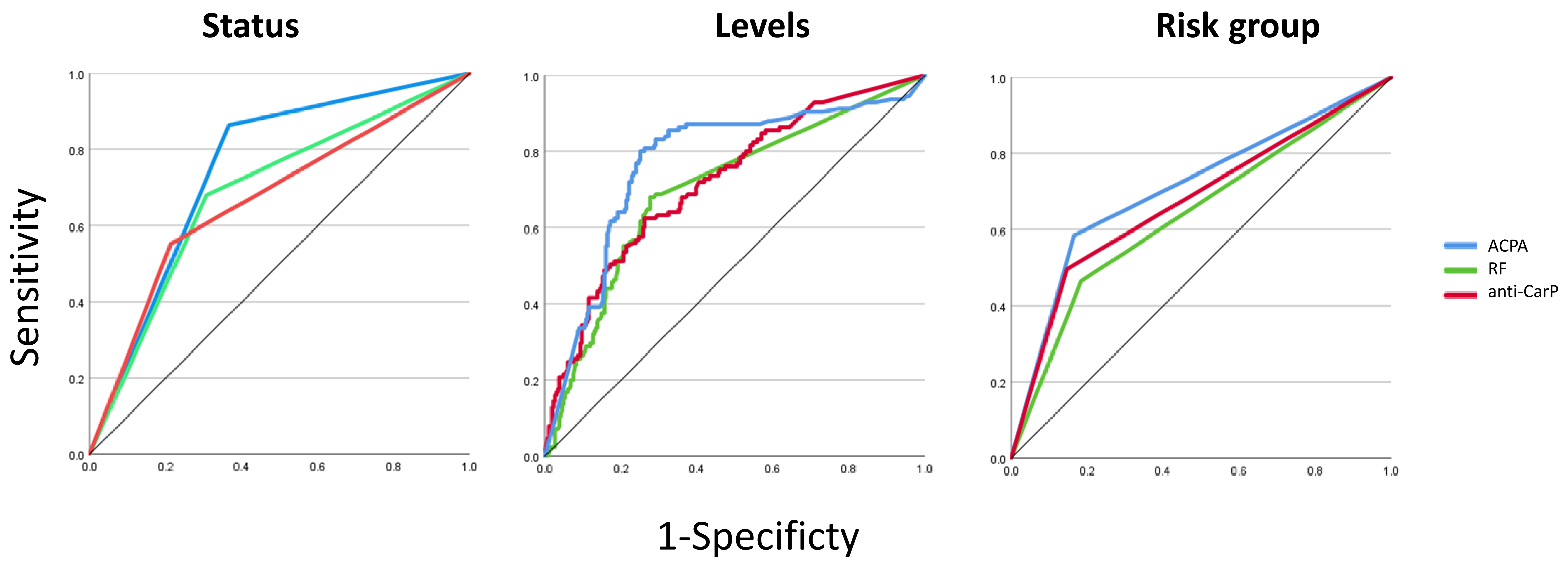

Supp-Figure 3 : Individual added value of the autoAbs over the clinical data only model (AUC)

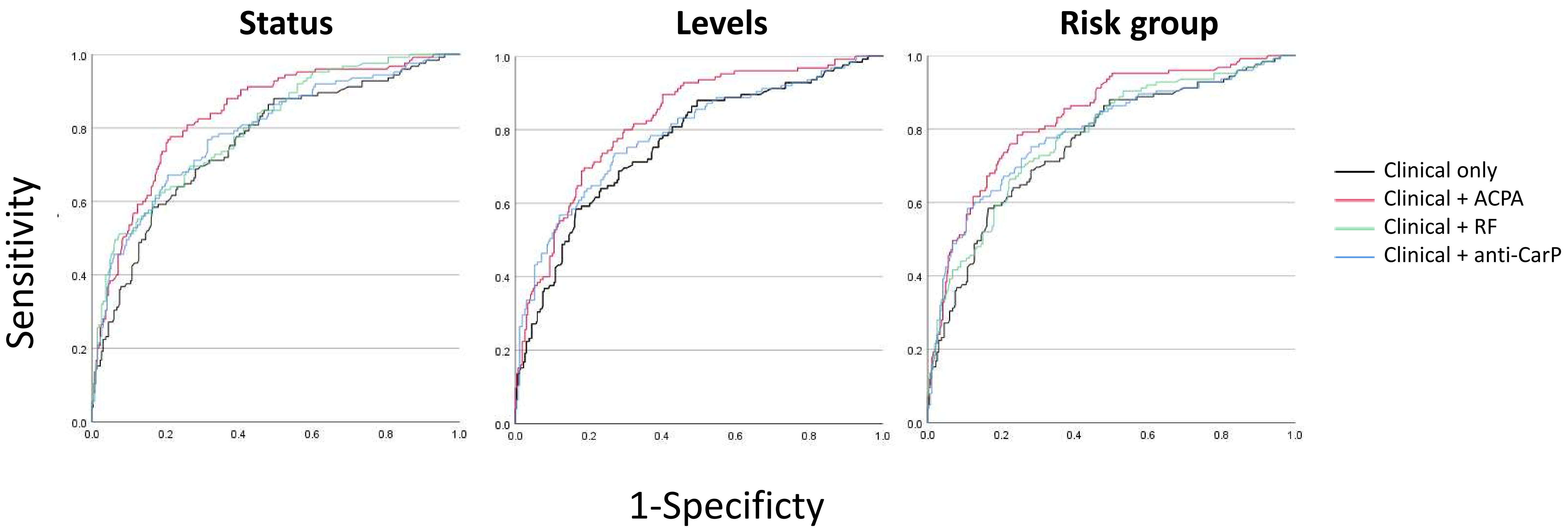

Supp-Figure 4 : Progression rate in groups of patients with different status for 3 autoAbs (ACPA/RF/anti-CarP)

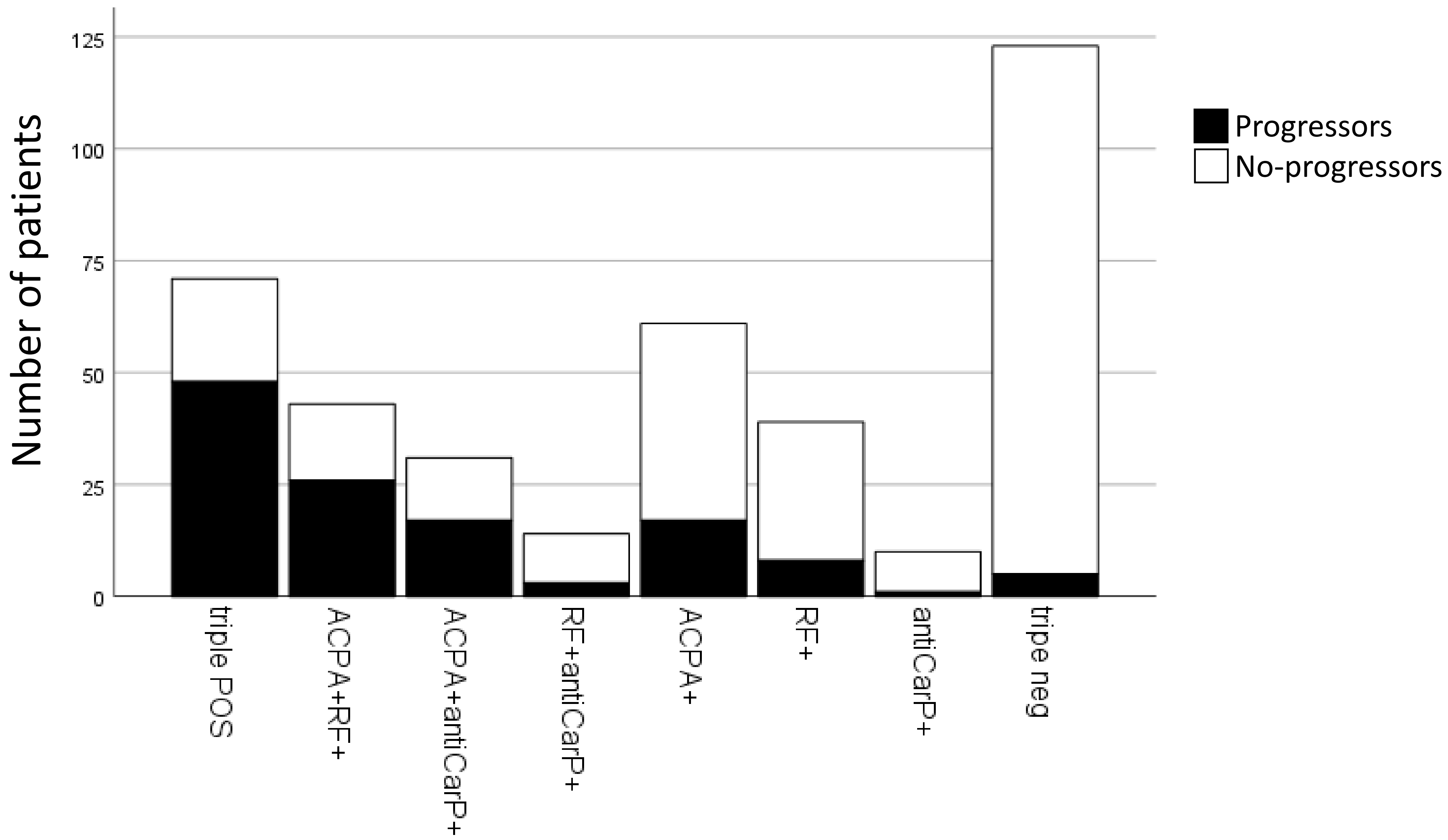

Supplement: Supplementary data [file rmdopen-2022-002512supp001.pdf]
